# Research Advances for Protein-Based Pickering Emulsions as Drug Delivery Systems

**DOI:** 10.3390/pharmaceutics17050587

**Published:** 2025-04-30

**Authors:** Long Deng, Junqiu Liao, Weiqi Liu, Xiaoxiao Liang, Rujin Zhou, Yanbin Jiang

**Affiliations:** 1 Guangdong Provincial Key Lab of Green Chemical Product Technology, School of Chemistry and Chemical Engineering, South China University of Technology, Guangzhou 510640, China; 2 School of Materials Science and Engineering, Guangdong University of Petrochemical Technology, Maoming 525000, China; 3 School of Chemical Engineering, Guangdong University of Petrochemical Technology, Maoming 525000, China

**Keywords:** protein, Pickering emulsion, interfacial properties, DPD simulations, drug delivery

## Abstract

Nanotechnologically engineered protein-based carriers have attracted considerable attention in the pharmaceutical field due to the advantages of superior biocompatibility, tunability and good emulsifying properties. Recently, protein-based Pickering emulsions (PPEs) systems with multi-level structures have been introduced as innovative colloidal delivery systems for advanced drug encapsulation, protection, delivery and controlled release. Natural source protein nanoparticles are promising candidates to provide a wide range of functional performances and interfacial properties in the preparation and stabilization of Pickering emulsions. Herein, this review summarizes the development of PPEs in drug delivery systems, focusing on the research progress concerning the aspects of protein particle preparation methods, formation mechanisms and rational design principles, emphasizing the relationship between protein particle structure and functional properties. To further understand the interfacial behavior in protein nanoparticle stabilized emulsion, the mesoscopic dissipative particle dynamics (DPD) simulations were discussed, which bridges the gaps between macroscopic time and length scales, as well as molecular-scale simulations on particles and oil/water interface systems. The structure-effect relationship between the tunable physicochemical properties of protein-based interface design, which leads to the effective loading, stimuli-responsiveness for the controlled release and multiple delivery, was then summarized. Finally, the opportunities and challenges for the future development of PPEs for drug delivery are discussed. This review aims to provide a reference for the further application of PPEs as advanced drug delivery systems.

## 1. Introduction

The development of drug delivery systems (DDS) holds significant promise for improving drug safety and therapeutic efficacy [1]. These systems aim to strategically encapsulate drugs to enhance protection, enable targeted delivery and facilitate controlled release through the rational design of DDS [2,3,4,5]. Driven by the micro/nanotechnology revolution, the DDS plays a prominent role in changing metabolic behavior in vivo, overcoming the multiple physiological barriers to promote drug absorption, which increases bioavailability, improves therapeutic efficacy and reduces toxic side effects [6,7,8,9]. Except for ensuring safe and efficient drug delivery, the delivery systems should incorporate green engineering technology and carriers which prioritize biocompatibility and bio-degradability to minimize toxicity to living organisms [10,11]. Various micro/nanoscale carriers and structures have been explored for DDS to achieve high stability, bioavailability, biocompatibility and delivery efficiency [12,13,14,15], including, but not limited to, inorganic nanoparticles, organic polymer particles, hydrogel capsules and lipid-based DDS (such as liposomes, emulsions and lipid particles) [16,17,18,19]. There are still certain limitations of conventional DDS, which have shown less effective drug loading and targeting [20], and biocompatibility limitations have restricted the application of inorganic materials for drug delivery [21]. The novel smart drug delivery system based on biomass carriers has been advanced to sense complicated micro-environments and work in concert with drugs to deliver one or more drugs precisely and intelligently [22].

During drug delivery, bio-interactions occurred mainly at the biointerfaces, which significantly influenced the in vivo fate of drugs and ultimately determined their delivery efficiency and biological effect [23,24]. Pickering emulsions, stabilized by colloidal particles adsorbed at the oil–water interface to form a robust physical barrier resisting droplet coalescence and Ostwald ripening [25], have shown great potential in the development of DDS by providing the interfacial particulate surface for drug release and bio-interactions [26,27]. It consists of an oil core and/or internal aqueous phase, oil–water interface and absorbed particles, which can be strategically loaded with several drugs of various physicochemical properties, such as different sizes, hydrophobicity or charges [28]. Particularly, it is the particles anchored to the interfacial layer that determined the properties of the Pickering emulsion, such as the formation of multi-level structure, high stability, multiple structure encapsulation, stimulus responsiveness and multifunctional physicochemical properties [29,30,31,32]. For biomedical and pharmaceutical applications, the use of emulsifiers with the potential for bespoke modification is typically based on inorganic or organic particles, such as silica or polymer-based systems [33,34]. With the focus on biocompatibility of the particle stabilization, there has been a drive to implement naturally-occurring proteins whose diversity, structure, biocompatibility, ability to form hierarchical self-assembly ranging from the nano to meso-scale, exquisite tunability and scalability, and lack of toxicity are attractive for the development of biologically relevant Pickering emulsions [35,36,37,38].

A variety of colloidal particles derived from different proteins have been identified for their ability to form and stabilize Pickering emulsions for drug delivery applications. For example, Li et al. [39] developed a pH-responsive intestinal-targeted Pickering emulsion delivery system stabilized by functionalized zein nanoparticles, which enhanced the bioaccessibility of curcumin. Similarly, Liu et al. [40] observed that complexes of oat protein isolate and high methoxyl pectin could create a dense interfacial film at the oil–water interface to improve the stability of the Pickering emulsion, which controlled the release of curcumin within a simulated gastrointestinal tract and improved bioaccessibility. By tuning the properties of protein particles and the absorptive interface, it is possible to tailor the stabilization and stimulus responsiveness of these systems for the controlled release of delivered drugs or components.

Recent literature has extensively discussed the formation, stability, properties and applications of protein-stabilized Pickering emulsions in the food industry [41,42]. Global patent databases (e.g., WIPO, EPO, CNIPA) have recorded numerous patents on protein-based Pickering emulsion technology for applications in food and pharmaceuticals. These patents encompass essential processes such as protein modification, particle size regulation and development of composite stabilizers. Notable examples include improvements in high-pressure homogenization equipment (WO2024187973A1) and advancements in the 3D-printed food molding technology (CN113973974A). This trend indicates that the technology is progressing from laboratory research toward industrial application. To achieve the efficient delivery and controlled release of the encapsulated drugs, it is crucial to gain a comprehensive understanding of the interfacial characteristics imparted by protein particles. This involves rationally adjusting particle sizes, surface charges, interfacial wettability and stimuli-responsiveness. While significant advances have been made in Pickering emulsions, it appears that a systematic review focusing on drug delivery systems utilizing protein-particle stabilized formulations is necessary. Furthermore, the stabilization mechanism through the regulation of the interfacial behavior of protein particles, as revealed by mesoscopic DPD simulations in conjunction with macroscopic experiments, remains inadequately explored. This gap significantly impedes the in-depth understanding of the adsorption properties of protein particles, the structure-effect relationship between their tunable physicochemical properties, and the efficiency of the delivery process.

In this paper, we aim to provide a comprehensive review of the research progress for protein-based Pickering emulsions (PPEs) in DDS. First, this review detailed the advancements in protein particle influencing factors and preparation methods, with stabilization mechanisms of Pickering emulsions, with particular emphasis on the correlation between the structure and functional properties of protein particles. To enhance the understanding of the interfacial behavior in protein particle-stabilized emulsions, the application of mesoscopic dissipative particle dynamics (DPD) simulation, which bridges the gaps between macroscopic time and length scales, as well as molecular-scale simulations on particles and oil/water interface systems, are discussed. The structure-effect relationship between the tunable physicochemical properties of protein-based interfaces and their delivery properties is summarized, along with their bioeffects in strategic encapsulation, efficient delivery and controlled release for the potential applications in drug delivery. Finally, the opportunities and challenges for the future development of PPEs for drug delivery were discussed.

## 2. Protein-Based Particles Stabilized Pickering Emulsion

### 2.1. Influence of Protein-Based Particle Properties on Stabilization of Pickering Emulsions

**Figure 1:**
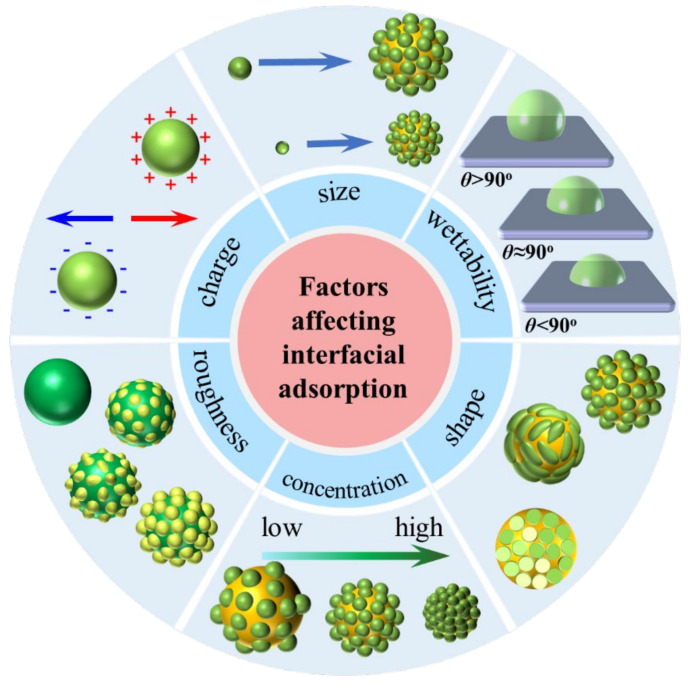
Schematic diagram of common influencing factors in PPE stabilization.

The effectiveness of protein-based particles in stabilizing Pickering emulsions is influenced by various factors, including particle wettability, size, charge, shape, roughness and particle concentration (Figure 1), which impact the particle interfacial adsorption at the oil–water interface [43,44,45,46,47,48,49,50]. Furthermore, other influencing parameters such as the oil phase type and ratio, salt concentration and aqueous pH present an important role in Pickering emulsion stabilization [51,52,53,54,55]. During the formation of Pickering emulsions, the protein particles need to be wetted by both oil and water in order to adsorb at the oil/water interface, and form a strong mechanical barrier against coalescence, oxidation and Ostwald ripening, resulting in Pickering emulsions with excellent stability. In addition, the electrostatic repulsion and steric repulsion between protein particles on the emulsion droplets also affect the flocculation, coalescence and Ostwald ripening, further resulting in improvement of the stability of the Pickering emulsions. The adsorption of particles at the interface is a key parameter for Pickering emulsion stabilization. The desorption energy of typical spherical particles from the interface strongly depends on the surface wettability, which can be expressed by the following equation [56]:

(1) $$∆E=\pi R^{2}{\left(1-|cos\theta |\right)}^{2}\times \gamma_{ow}$$

where ∆*E* is the energy required to move the particles out of the interface, *R* is the radius of the spherical particles (m), *γ_ow_* is the interfacial tension between the oil and water phases (N/m), and *θ* is the contact angle representing the surface wettability, respectively. To achieve the minimum energy, particles were expected to exhibit appropriate wettability, size and interfacial tension between the oil and water phases for emulsion stabilization [57,58,59].

The wettability of protein particles is the critical factor in the formation and stability of Pickering emulsions [60]. Extremely hydrophilic or hydrophobic protein particles are unable to fully adsorb at the oil–water interface, resulting in reduced stability of the Pickering emulsion. A previous study has reported that the modification of zein particles with water-soluble tannic acid and glycyrrhiza acid made them more hydrophilic, improving the wettability of the particles to achieve stable Pickering emulsion delivery systems [39]. The three-phase (disperse phase, continuous phase and the protein particles) contact angle (*θ*), which represents the particle wettability, could predict the interfacial behavior of the particles and it is a crucial property for the absorption of particles at the oil/water interface. In general, if the *θ* is less than 90 °, particles in the oil phase will be more wetting to stabilize O/W emulsions. On the other hand, if the *θ* is greater than 90°, the particle will be more present in the aqueous phase to stabilize W/O emulsion. To achieve durable emulsion stabilization, the particle wettability should be close to 90°. Multiple water-in-oil-water (W/O/W) and oil-water-oil (O/W/O) Pickering emulsions can also be stabilized using one or two types of protein particles [61,62]. The wettability of protein particles can be regulated through hydrophilic or hydrophobic modification of the particle surface by physical regulation or chemical modification [43]. In addition, the characteristics of the oil phase and the pH and ionic strength of the aqueous phase, significantly influence the wettability of protein particles [41].

Protein particle size also has a significant impact on the stabilization of the Pickering emulsions. According to Equation (1), larger particles require more energy to anchor at the oil/water interface with slower absorption kinetics and weaker packing efficiency over the droplet surface [63], while smaller particles allow a more stable particle droplet to be obtained due to the lower energy [60]. It has also been shown that particle size determines the emulsion droplet size [63], and an increase in the protein particle size is accompanied by an increase in the emulsion droplet size [56]. For PPEs, the size of the protein particles affects the absorption and accumulation at the oil–water interface, as well as the mobility of the protein particles in the continuous phase, the rheological properties, the stability and the texture properties of stable PPE droplets [64]. Generally, the smaller the average protein particle size of PPEs, the greater the stability of Pickering emulsions. The smaller the protein particle size, the more favorable the particle diffusion and absorption at the oil–water interface, thus promoting the formation of smaller droplets and enhancing the stabilization of Pickering emulsions. Also, a smaller average protein particle size results in a lower minimum particle concentration required for the emulsification system [65].

Furthermore, the protein particle surface charges also influence the formation and stability of Pickering emulsions [66]. The dissociation or deprotonation of functional groups on the surface of protein particles directly determines the surface charge, thus affecting their dispersion state in the dispersing solution [50]. Protein particles with high surface charge usually form a greater electrostatic repulsion force than those with low surface charge, which show better dispersion and form a stable state between particles [67]. Then the protein particles with higher surface charge exist in a single-layer arrangement on the surface of the oil droplet, effectively preventing the collision between oil droplets to form more stable Pickering emulsions. Sun et al. [68] reported that Pickering emulsion stabilized by higher charge hemp protein nanoparticles showed stable storage stability.

In addition, it has been reported that the repulsive force between protein particles and droplets would slow down the adsorption kinetics and increase the instability of emulsions with too high a surface protein particle charge [56]. Therefore, in the case of poor adsorption of particles at the oil–water interface, reducing the particle surface charge will improve the stability of the Pickering emulsion [69]. Most oil phases have negative surface charges when dispersed in water [70]. Thus, the cationic protein nanoparticle stabilizers could improve the Pickering emulsion stability due to the stronger affinity of the particles at the oil–water interface [71]. To achieve electrostatic and emulsion stability, the surface charge of protein particles can be tuned by adjusting the pH and salt ion concentration and by functional group modification [72].

Furthermore, the protein particle shape plays a vital role in the formation of PPEs. Non-spherical particles such as protein fibrils and protein tubes with different aspect ratios affect the particle adsorption and interfacial arrangement at the oil–water interface, resulting in stable emulsions [73]. The softness of protein particles induces particle–particle interactions and the adsorption mechanism compared to rigid particles [74]. The particle surface roughness also influences the wettability by affecting the contact surface, which affects the Pickering emulsion stability [75]. The protein particle concentration affects the PPE droplet size. As the particle concentration increases, the droplet size decreases homogenously; this process is called “limited coalescence” [76]. Whereas at low particle concentrations, the emulsion exhibits instability with large droplet size due to a lack of particles to stabilize the droplets [77].

Additionally, the particle concentration induces phase inversion and tuning of the single or multiple Pickering emulsion. Apart from modifying the protein particle properties, regulating the dispersion and continuous phase is another strategy to stabilize the Pickering emulsion. It has been reported that the polarity and viscosity and the ratio of the oil phase induce the alteration of the contact angle, and thus influence the emulsion droplet size, type and stabilization [56]. Additionally, the salt concentration and pH, which ionize the surface groups of particles, also significantly affect the particle surface charge and the contact angle to increase the Pickering emulsion stability [25].

It is crucial to note that the preparation method of Pickering emulsions also influences their stability. Due to the high interaction energy at the interface, Pickering emulsions require external forces to overcome the substantial energy barrier associated with particle adsorption. High-energy input methods, such as homogenization and sonic emulsification [78], effectively break large oil droplets into smaller ones, making them suitable for large-scale production. However, these methods often result in a non-uniform droplet size distribution. Additionally, high mechanical shear may induce protein particle aggregation or lead to emulsion instability, complicating the preparation of submicron emulsions (<1 μm). In contrast, monodisperse emulsions can be achieved through low-energy, controlled techniques such as membrane emulsification and microfluidics [26]. Microfluidics facilitates precise regulation of droplet properties with high uniformity by manipulating parameters such as the two-phase flow rate ratio and mixing rate, which is critical for controlled release applications [79]. Therefore, optimizing both particle properties (size, wettability, charge) and process conditions (pH, ionic strength) is essential for achieving a balance between emulsion stability and delivery efficiency.

Phase diagrams can be employed to determine the optimal emulsification region for multicomponent emulsions, with a significant focus on conventional surfactant-stabilized microemulsion systems [80,81,82,83,84]. However, there is a notable lack of phase diagram studies concerning protein-based Pickering emulsifiers. This gap may arise from the complexities of Pickering emulsions as multicomponent colloidal systems, which require consideration of particle wettability, size, shape and interfacial adsorption, making it challenging to accurately determine phase boundaries. Most related studies have prioritized specific formulation optimization over comprehensive phase behavior analysis [85]. In the future, the phase behavior of complex Pickering emulsions is expected to be investigated through the integration of molecular dynamics simulations. This approach will enable a thorough analysis of the influences of protein particle properties and preparation conditions on Pickering emulsion.

In summary, it is imperative to consider all the important parameters associated with the protein particles, such as size, charge, morphology, pH, concentration and adsorption at the oil–water interface which are interrelated and influence the particle wetting properties. Although these factors allow the tuning of PPE properties and characteristics to meet the requirements of the drug delivery application, it is very complicated to study the contribution independently. The different surface chemical compositions of protein particles could lead to different wetting properties and amplification. By rationally regulating the parameters of protein particles, it is beneficial to obtain a PPE DDS with properties such as biocompatibility, versatility and excellent stability.

### 2.2. Preparation and Modification of Protein-Based Particles for Pickering Emulsions

Protein-based particles can be derived from natural sources or synthesized to stabilize Pickering emulsions. Given the diversity of proteins in terms of the chemical structure and conformation, their solubility and aggregation states can be modulated by factors such as solvents, temperature, ions and pH, involving both covalent and non-covalent interactions, such as disulfide bonds, hydrogen bonds, hydrophobic interactions and steric repulsions. From the perspective of protein interactions, particle preparation methods include physical, chemical, and biological approaches that allow the formation of particles capable of stabilizing emulsions with different physicochemical properties [43]. Table 1 provides a comprehensive overview of these methods, offering a concise summary, evaluation and critical analysis.

Despite the growing interest in the development of protein-based particle emulsifiers, the large-scale application of these particles in stabilizing Pickering emulsions remains challenging due to their inherent limitations. These limitations include poor water solubility, relatively low digestibility, weak emulsifying activity, lack of responsiveness to stimuli and instability under physiological environmental conditions (such as pH, temperature, ionic strength and enzyme) in their native state [99]. The key parameters of Pickering emulsions stabilized by various proteins are summarized in Table 2, including preparation method, quantitative emulsification criteria and stability performance. These results elucidate the structure-function relationship between protein properties and emulsion stability, offering critical insights for the design of a drug delivery system. To enhance the emulsifying and stabilizing properties of protein-based particles, particularly regarding the biocompatibility and bio-degradability of PPE delivery systems, the modification of proteins with bio-based materials, such as polysaccharides and polyphenols, has recently emerged as an attractive strategy [100,101]. As summarized in Table 3, these biologically derived ligands facilitate the assembly, and design of complex protein Pickering particle emulsifiers with tunable wettability and stimuli-responsive functionalities.

## 3. Stabilization Mechanism and Interfacial Properties of PPEs

**Figure 2:**
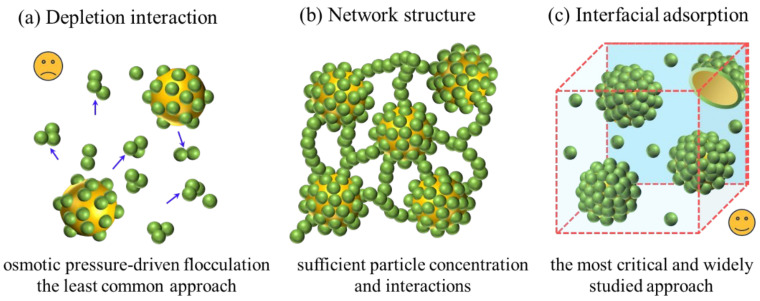
Schematic representation of the stabilization mechanisms of PPE: (**a**) depletion interaction stabilization mechanism, (**b**) network structure stabilization mechanism, (**c**) interfacial adsorption stabilization mechanism. Reproduced with permission from [50]. Copyright @2024 Elsevier.

It is well known that the stabilization mechanism of Pickering emulsions involves the particle emulsifier adsorbed at the oil–water interface inhibiting emulsion droplets from destabilizing by forming a mechanical barrier [111]. Unlike conventional rigid inorganic particles, protein particles perform more like soft particles and exhibit deformable properties at the oil–water interface. Recent studies have extensively investigated the stabilization mechanism and interfacial properties of PPEs, identifying three primary mechanisms: depletion, particle network structure formation, and interfacial particle adsorption stabilization [41], as shown in Figure 2.

Depletion stabilization, the least common approach, utilizes non-adsorbed proteins to induce osmotic pressure-driven flocculation, thereby enhancing emulsion stability [50]. The network structure stabilization mechanism relies on the formation of a three-dimensional viscoelastic network of non-adsorbed particles, requiring both sufficient particle concentration and attractive interactions [112]. The most critical and widely studied mechanism is the interfacial stabilization mechanism, where protein particles with appropriate wettability irreversibly adsorb at the oil–water interface, forming a dense barrier that prevents droplet coalescence through volume repulsion and relies on the formation of a thick interfacial layer of high-energy adsorbers [72,113]. The stability of PPEs is predominantly determined by the adsorption and interfacial properties of this dense interfacial layer, encompassing its formation, the dynamic adsorption process, and the arrangement and deformation of particles at the interface [50].

PPEs demonstrate enhanced stability compared to conventional surfactant-based emulsions, primarily due to the establishment of a particle-rich interfacial region rather than a mere geometric interface. The properties of protein particles, including wettability, size and charge, as discussed in Section 2.1, play a significant role in the formation of the interfacial layer. During the formation of PPEs, the dynamic adsorption of protein particles is critical for emulsion stability, with optimal adsorption rates preventing aggregation, while slower adsorption rates lead to destabilization and decreased homogenization efficiency [50]. As protein particles continuously adsorb at the interface until saturation is reached, they may undergo rearrangement and deformation influenced by surface density, repulsive forces and capillary interactions [114,115,116]. In the protein particle-rich state, high surface density and repulsive interactions prevent droplet coalescence, and promote the formation of gel-like network structures. These factors also impact interfacial deformation and particle stacking, subsequently affecting conformational changes that reduce elastic energy and enhance emulsion stability through lateral capillary attraction [111].

The formation and stability of PPEs are influenced by factors such as particle size, wettability, concentration and particle adsorption, thus requiring tailored approaches for optimal performance in pharmaceutical applications.

### 3.1. Macroscopic Experimental Techniques on Interfacial Properties

**Figure 3:**
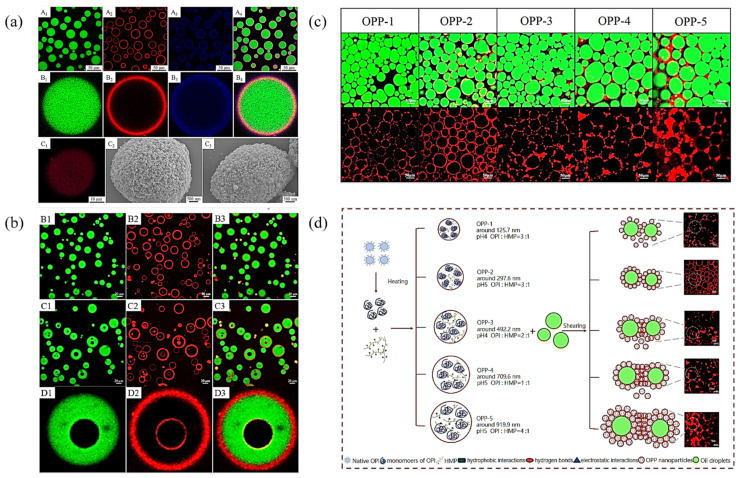
Application of SEM and CLSM to characterize the morphology, interfacial particle formation (**a**–**c**) and the formation mechanism (**d**) of Pickering emulsions. (**a**) CLSM images of ZTG-stabilized Pickering emulsions (O/W) at a ZTG concentration of 0.5% (**A1**–**A4**) and its magnified images (**B1**–**B4**). Corn oil was stained with Nile Red (green), zein was stained with Nile Blue A, and GA was stained with thioflavin T (blue). (**C1**) 3D-reconstructed CLSM image of ZTG-stabilized Pickering emulsions. (**C2**) SEM images of Pickering emulsions at a ZTG concentration of 0.5% and (**C3**) 2.0% *w*/*v*. Reproduced with permission from [39]. Copyright @2023 American Chemical Society. (**b**) CLSM images of ZA NPs (**B1**–**B3**) and ZAZn NPs (**C1**–**C3**) stabilized Pickering emulsions (W/O/W) and its magnified images (**D1**–**D3**). Corn oil was stained with Nile Red (green), and zein was stained with Nile Blue A (Red). Reproduced with permission from [71]. Copyright @2025 Elsevier. (**c**) CLSM images of Pickering emulsions stabilized by OPP with different particle sizes. The green and red regions represent the oil droplets (green) and the OPP, respectively. OPP were strained by Rhodamine B (red) excited at 533 nm. (**d**) The formation mechanism of OPP and OPP-stabilized Pickering emulsions. Reproduced with permission from [40]. Copyright @2024 Elsevier.

**Figure 4:**
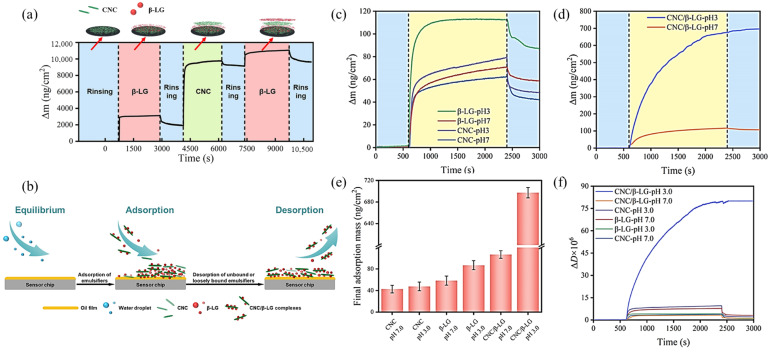
Application of QCM-D to investigate the adsorption mass and dissipation characteristics of protein particle interfacial adsorption. (**a**) Adsorption properties of β-LG (pH 3.0) onto the CNC-coated surface. (**b**) Schematic diagrams of CNC/β-LG complexes at the corn oil-coated surface. (**c**–**f**) Adsorbed mass (Δm) and dissipation (ΔD) from the fifth overtone as a function of time for CNCs, β-LG and CNC/β-LG complexes at pH 3.0 and pH 7.0 during adsorption and rinsing at the corn oil-coated surface. Reproduced with permission from [106]. Copyright @2025 Elsevier.

The formation and stability of PPEs primarily depend on the interfacial properties of the protein particles and the interfacial layer, thus characterizing the properties and behavior of protein particles at the oil–water interface across multiple scales, is crucial for elucidating the mechanism underlying the formation and stabilization of PPEs. We briefly review and evaluate various macroscopic experimental techniques employed to qualitatively or quantitatively characterize Pickering emulsion morphology, interfacial film visualization, interfacial tension, interfacial adsorption mass, thickness and interfacial rheology [117]. For example, CLSM is frequently employed to determine the type of Pickering emulsion, the localization of particle adsorption, and to visually monitor the thickness of the particle adsorption layer, while SEM is used to confirm the formation and adsorption thickness of interfacial particles, thereby facilitating a deeper analysis of the emulsion formation mechanism [39,40,71] (Figure 3). Quartz crystal dissipation microbalance (QCM-D) emerged as a valuable tool for real-time monitoring of particle adsorption behavior at the oil–water interface, providing quantitative data at the nanogram scale [118]. Recent studies, such as those by Wu et al. [106], utilized QCM-D to investigate the adsorption mass and dissipation characteristics of cellulose nanocrystal/β-lactoglobulin complexes, offering insights into the nature and viscoelastic properties of oil–water interfacial membranes, and enhancing the understanding of the interactions between protein complexes and oil–water interfaces (Figure 4).

It is important to acknowledge that interfacial properties derived from different measurement methods may differ significantly due to the varying principles of these techniques and the inherent limitations of interface models used to simulate real emulsion systems. Nevertheless, results obtained from the same measurement method remain valid and comparable. A summary of the advantages and limitations of the various techniques is presented in Table 4.

### 3.2. Mesoscopic DPD Simulations for the Stabilization Mechanism

Due to the multi-scale and complex nature of PPEs, establishing a suitable and reliable oil–water interface model that accurately and non-destructively reflects the real emulsion systems remains a challenge. In contrast to traditional macroscopic experimental techniques, molecular dynamic (MD) simulations, which are based on atomistic methods, intuitively capture the structural changes of emulsifier molecules during diffusion, penetration and reorganization [119]. This offers significant advantages for studying the adsorption and desorption processes of emulsifier molecules at the oil–water interface. However, MD simulations are limited in their ability to address phenomena involving more than a million atoms [120].

**Table 4:** Different interfacial techniques for Pickering emulsions stabilized by protein particles.

Interfacial Properties	Techniques	Advantages	Limitations	References
Visualization technology of morphology and the interfacial film	Optical microscopy	Simple operation, quick view of the structure and integrity	Hard to characterize the Pickering emulsion type and in-depth analysis of the morphology	[121]
	Scanning electron microscopy (SEM)	Observe the structure and morphology of the Pickering emulsion, assess the thickness of the Pickering shell formed by the solid particles, analyze in detail the adsorption behavior of particles	Cumbersome operating procedure, Pickering emulsion requires polymerization or freeze drying and gold/platinum coating	[39,122]
	Confocal laser scanningmicroscopy (CLSM)	Reveal and structure and location of the interfacial adsorbed particles, analysis of the emulsion type, visual on the cross-sectional layer of a Pickering emulsion, 3D reconstruction	Tedious stain procedure dependent on target particles, continuous phase, dispersed phase and the staining agent	[123]
	Transmitting electron microscopy(TEM) and Cryo-TEM	High definition and high resolution to detect the interfacial adsorbed particle, nanoscale monitoring of the cross-sectional image	Tedious operating procedure, needs special sample holder (e.g., copper grid)	[124]
	Atomic force microscopy (AFM)	Measure the stiffness and strength of the interfacial shell based on tip-surface interaction and atomic-level resolution and detect the adsorbed particle, analysis of competitive adsorption and morphology	Complex sample preparation, limited to static films, special modifications to the probe tip may be required for stiffness and interface shell strength measurements, poor image quality	[125]
	Brewster Angle Microscopy (BAM)	In situ dynamic monitoring of interface structures, no labeling required	Lower resolution (μm-scale), limited to air-water or oil–water interfaces	[126]
Interfacial tension	Pendant drop	High precision, real-time calculates γ via Young-Laplace equation based on droplet shape, suitable for static/dynamic adsorption monitoring	Sensitive to droplet size, requires optical calibration	[127]
	Wilhelmy plate	Measures vertical force on a platinum plate immersed in liquid to calculate γ via contact angle, simple operation, wide γ range	Susceptible to surface contamination	[128]
	Microfluidics	High-throughput, monodisperse droplet control, combines droplet generation in microchannels with optical imaging to calculate γ	Expensive setup, precise flow control required	[129]
Interfacial adsorption mass and thickness	Centrifugation	Simple operation, separation of serum and cream layers, adsorbed mass derived from surface coverage and assumption of equilibrium partitioning	Underestimation for small droplets; lacks real-time dynamic monitoring	[130]
	Polystyrene Latex Particles	Indirect measurement via particle size changes due to adsorbed layers, quantifies thickness in model systems, mimics emulsion environments	Simplified models may not capture complex interfacial dynamics	[131]
	Film Interferometry	High-precision in situ measurement, dynamic monitoring, interference patterns correlate with film thickness and refractive index	Requires transparent systems; complex optical alignment	[132]
	Ellipsometry	Non-destructive, high resolution, real-time capability, Fresnel equations relate polarization changes to interfacial properties	Sensitive to surface roughness, requires complex modeling	[133]
	Quartz Crystal Microbalance with Dissipation (QCM-D)	High sensitivity, real-time adsorption kinetics, structural insights, frequency (Δf) and dissipation (ΔD) shifts used to calculate mass and viscoelasticity	Expensive sensors, simplified models may ignore interfacial complexity	[106]
Interfacial rheology	Interfacial dilatational rheology (Langmuir Blodgett)	Investigate the dynamics of the adsorption process of particles at the interface (diffusion, penetration, recombination) and the viscoelastic modulus of the interface film (elastic modulus Ed, viscous modulus Ev) via Langmuir trough or pendant-drop methods	Limited frequency range, only applicable to low frequencies	[134]
	Interfacial shear rheology	Investigate the mechanical behavior of interfacial films under shear forces (elastic modulus, G′ viscous modulus, G″) to reveal intermolecular interactions and network structures, compatible with stress/strain-controlled rheometers, versatile testing modes	Low sensitivity for small deformations	[49]

Recently, dissipative particle dynamics (DPD) based on mesoscale coarse-grained methods, has been introduced to model colloidal emulsion systems, bridging the gap between macroscopic time and length scales and molecular-scale simulation. Ahmadi et al. [135] reviewed the fundamentals of DPD and its applications to surfactant-water-oil mixture colloidal and interfacial systems. DPD simulates clusters of atoms or molecules by coarse-graining the molecular interactions into “beads”, allowing the capture of dynamic behavior over larger temporal and spatial scales. The forces in DPD include conservative, dissipative and random components, ensuring thermodynamic consistency and hydrodynamic behavior [136]. Consequently, DPD is also suitable for the study of PPEs, particularly with regard to the adsorption and self-assembly processes of emulsifier particles at the oil–water interface.

Fan et al. reported a mesoscale DPD simulation method for analyzing the droplet coalescence of Pickering emulsions, effectively circumventing the limitations associated with traditional experimental techniques [137]. DPD simulations facilitate the multi-scale modeling of composite Pickering emulsion systems, enabling the observation of the formation and stabilization processes of emulsion droplets [138,139]. In addition, DPD is particularly adept at studying the interfacial dynamic adsorption behavior of Pickering emulsions, elucidating interfacial phenomena, particle behavior, and their subsequent influence on emulsion stability in a highly controlled virtual environment.

**Figure 5:**
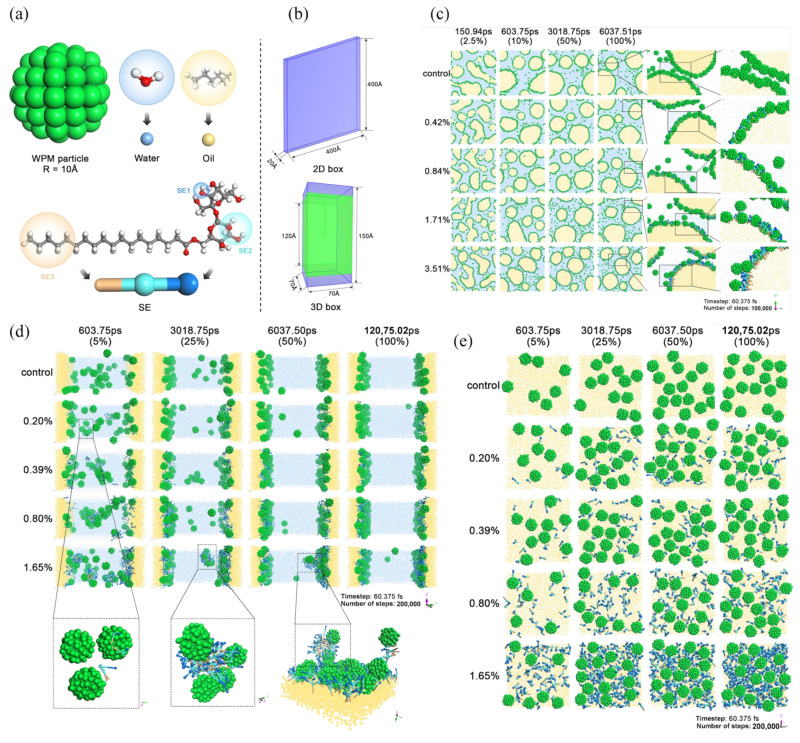
Application of DPD simulations to examine the dynamic adsorption and stability mechanisms of protein particle-stabilized Pickering emulsions. (**a**) Coarse-grained modeling of WPM, hydrophilic sucrose esters, water, and triglyceride; (**b**) 2D and 3D boxes. (**c**) Snapshots of DPD trajectory for different SE concentrations (0, 0.42 mol%, 0.84 mol%, 1.71 mol%, and 3.51 mol%) of CPE at different simulation durations in 2D box. (**d**) Snapshots of DPD trajectory for different SE concentrations of CPE at different simulation durations in 3D box. The callout boxes indicate the morphology of WPM and SE in the bulk phase at low SE concentrations (0.20 mol%), the morphology of aggregates formed in the bulk phase at high SE concentrations (1.65 mol%), and the morphology of aggregates adsorbed at the interface. (**e**) Stacking patterns of emulsion interfacial components on the same area (70 × 70 A2) of the oil–water interface at different SE concentrations. Reproduced with permission from [140]. Copyright @2025 Elsevier.

As shown in Figure 5, Ye et al. [140] used DPD simulation in both two-dimensional and three-dimensional boxes to investigate the dynamic adsorption and stability mechanisms of whey protein microgel particles (WPM) and sucrose esters (SE) in co-stabilized Pickering emulsions. The DPD simulations focused on morphological evolution, interfacial layer compactness, near-interface diffusion of components and interfacial barrier thickness. The results showed that higher SE concentrations led to increased interfacial density and compactness at the oil–water interface, driven by SE adsorption and WPM anchoring, which ultimately improved film stability. The post-equilibrium rearrangement resulted in a densely packed structure with SE occupying the spaces between WPM. This observation was consistent with the experimental results, and confirmed an optimal SE concentration maximized protein presence and interfacial stability.

Although DPD also has limitations, such as the necessity for careful parameterization of bead interactions and challenges in accurately modeling high-temperature and high-pressure conditions relevant to industrial applications [136], it has been effectively used to study and understand the mesoscopic dynamics of Pickering emulsions. DPD provides valuable insights into emulsifier adsorption, interfacial film formation and emulsion stability, thus it is especially potentially valuable in explaining the adsorption behavior and stabilization mechanisms of complex Pickering emulsions (e.g., W/O/W). Future improvements in parameterization and integration algorithms will further enhance its applicability to real PPE systems.

## 4. Applications of PPEs in Drug Delivery Systems

Through nanotechnological engineering of protein particles for optimized assembly and adsorption at the oil–water interface, PPEs exhibit high stability, multi-level structures and tunable functionality. Recently, studies on PPEs have attracted considerable attention in the context of pharmaceutical applications, particularly for encapsulation and drug delivery. Pharmaceutical Pickering emulsions are primarily used in topical and oral drug delivery systems [11,141], with comparatively less focus on injectable formulations due to challenges related to droplet size and osmotic pressure instability [142]. For this reason, in this section we will focus exclusively on summarizing the encapsulation, delivery and release of PPEs in oral drug delivery.

### 4.1. Drug Encapsulation

**Figure 6:**
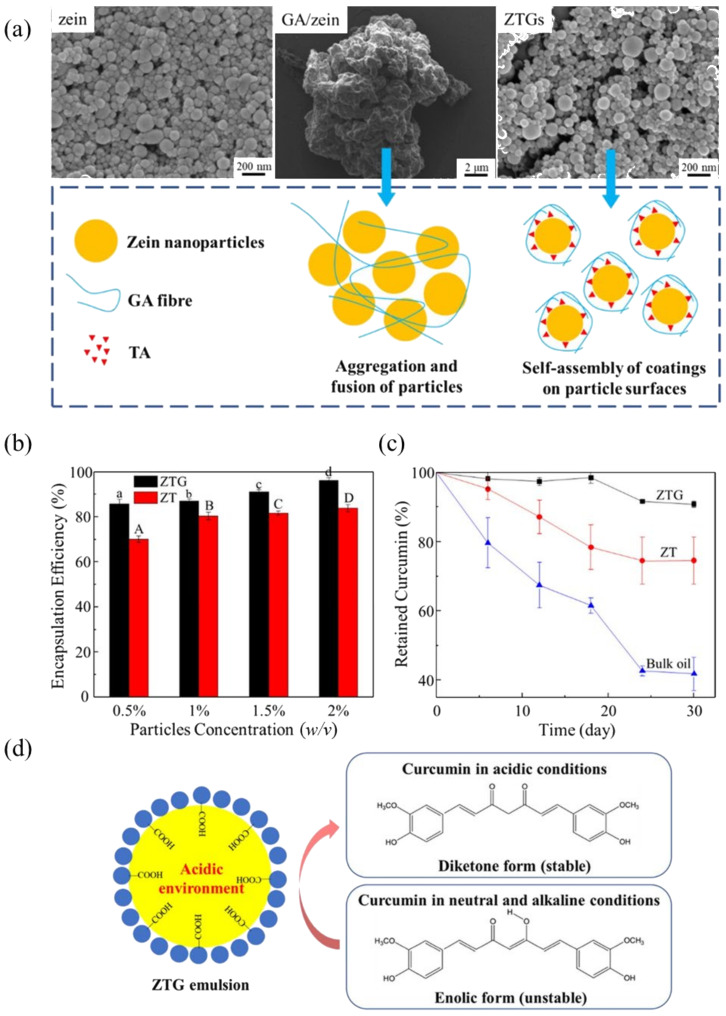
(**a**) SEM images of zein nanoparticles, GA/zein, and ZTGs. (**b**) Encapsulation efficiency of curcumin in ZTG-stabilized and ZT-stabilized emulsions with different particle concentrations. Different letters indicate significant differences (*p* < 0.05). (**c**) Retained curcumin in ZTG-stabilized, ZT-stabilized emulsions, and bulk oil for 30 days of storage. (**d**) Schematic diagram of higher encapsulation efficiency of curcumin in ZTG emulsion. Reproduced with permission from [39]. Copyright @2023 American Chemical Society.

Drug encapsulation has emerged as a recognized and effective strategy for enhancing stability, solubility and bioavailability [143]. For successful drug encapsulation, PPEs must fulfill several critical requirements, including compatibility with the drug, exceptional physical stability to protect the drug from the external environment, high drug loading and retention capacity, and responsiveness to external stimuli to facilitate the drug release [41,144]. PPEs stabilized by interfacial protein particles provide a versatile platform for the strategic drug loading of hydrophobic drugs within oil nuclei through hydrophobic interactions, chemical linking, or electrostatic adsorption mechanisms. For example, Li et al. [39] achieved high encapsulation efficiency of curcumin in the O/W Pickering emulsion stabilized by zein-based nanoparticles, which were coated with glycyrrhizic acid (GA). By aggregating more particles at the interface to form a compact barrier, the degradation of curcumin is limited, resulting in increased encapsulation efficiency as particle concentration rises. In addition, this GA coating creates a favorable acidic micro-environment that enhances the stability of the curcumin molecules (Figure 6).

**Figure 7:**
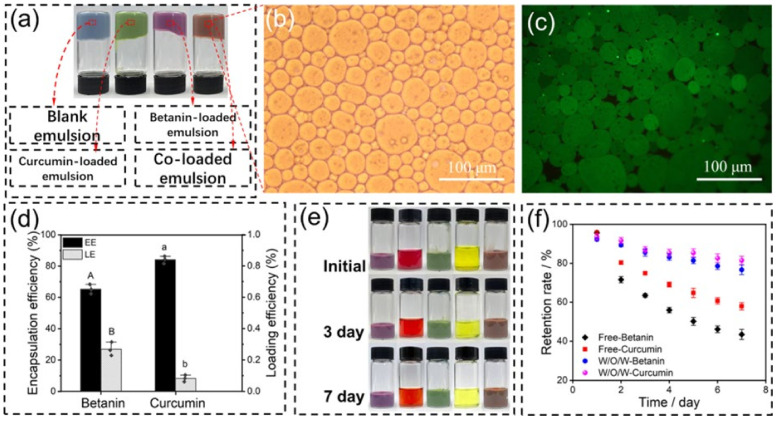
(**a**) Visual appearance of blank emulsions, betanin-loaded emulsions, curcumin-loaded emulsions, and curcumin and betanin-loaded emulsions. (**b**) Optical microscopy appearance of W/O/W droplets in the bright field mode. (**c**) Optical microscopy appearance of W/O/W droplets in the fluorescent field mode. (**d**) Encapsulation efficiency and LE of betanin and curcumin. (**e**) Visual appearance of different emulsions stored for 3 and 7 days at RT, from left to right: betanin-loaded emulsions, free-betanin, curcumin-loaded emulsions, free-curcumin, and co-loaded emulsions. (**f**) Percentage of remaining betanin and curcumin after various times of storage at RT with and without emulsification with the SBNPs. Bars with different letters (A and B, a and b) indicate statistically significant (*p* < 0.05) differences among samples. Data are mean ± standard deviation of the triplicates. Reproduced with permission from [145]. Copyright @2021 American Chemical Society.

Furthermore, the multi-level structure of multiple PPEs enables the co-encapsulation of multiple drugs with different hydrophilic and hydrophobicity, effectively addressing challenges associated with spatiotemporal delivery for applications such as cancer therapy. For example, Zhang et al. [145] developed a water-in-oil-in-water (W/O/W) Pickering emulsion stabilized by sugar beet pectin-bovine serum albumin nanoparticles, achieving efficient co-encapsulation of betanin (65.3%) in the inner water phase and curcumin (84.1%) in the oil phase, which resulted in synergistic anti-tumor effects (Figure 7). Additionally, in previous work [71] we successfully co-encapsulated curcumin and fluorouracil in a W/O/W Pickering emulsion stabilized by zein/anthocyanin (ACN)/Zn^2+^ particles, demonstrating a high curcumin encapsulation efficiency (91.2%) attributed to the complexation of Zn^2+^ with curcumin, thereby enhancing the anti-cancer activity of fluorouracil. These findings highlight the potential of PPEs as advanced multi-drug delivery systems for improved drug encapsulation efficiency, bioavailability and therapeutic efficacy.

### 4.2. Controlled Release

Oral drug delivery systems hold significant promise for enhancing drug stability, bioavailability and patient compliance, while they must overcome challenges of unpredictable adsorption of uncontrolled degradation by enzymes, microbial environments and gastric acid [146]. PPEs offer unique advantages for oral delivery due to their high surface areas and customizable surface functionality, which can be tailored to respond to the gastrointestinal environment. By modifying protein particles with bioactive functional components that exhibit environmental responsiveness, it is possible for PPEs to achieve targeted delivery and controlled release kinetics, addressing the need for site-specific and stimuli-triggered drug release. The drug release mechanisms in PPEs can be finely tuned through two primary strategies [26]: (1) leveraging the permeability of interparticle gaps to enable diffusion-driven release of encapsulated drugs, influenced by factors such as particle characteristics, interfacial properties, shell thickness, layer-by-layer deposition and diffusion coefficients; (2) designing stimuli-responsive interfacial particles that destabilize in response to external cues (e.g., pH, temperature or light), leading to droplet rupture and burst release. By balancing stability and responsiveness, PPEs can be engineered as advanced delivery systems for controlled and stimuli-triggered drug release, optimizing therapeutic outcomes in oral applications.

Diffusion-controlled release from PPEs is expected to exhibit a slow and constant release profile by managing particle size, variability, concentration, and concentration gradient between the continuous and dispersed phases, all of which affect permeability [26]. Additionally, thicker shells and layer-by-layer deposition contribute to a slower and prolonged release [26]. Compared to the diffusion-controlled release, stimuli-responsive release may provide insights into smart release mechanisms and remote control over the delivered drugs.

**Figure 8:**
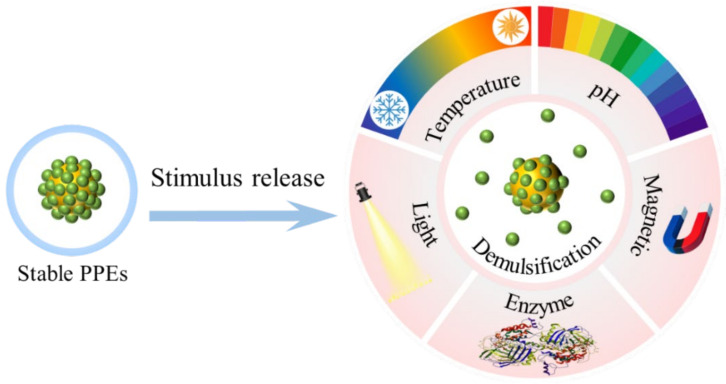
A graphical illustration showing the PPEs triggered release behavior.

As protein particle-assembled systems, PPEs inherited distinct physio-chemical properties from the adsorbed protein-based particles. They remain stable until an external trigger is applied, which can alter the wettability, charges, or sizes of particles, leading to disruption of the interfacial arrangement and facilitating emulsion breakage. This offers opportunities for precise control of drug delivery directly at the disease site. The design of PPEs selectively responds to environmental triggers such as pH, temperature, light, enzyme activity and magnetic fields (Figure 8) [146].

pH responsiveness is one of the most widely investigated release mechanisms in stimuli-responsive oral drug delivery systems, leveraging the pH variations present in the target tissue, such as the acidic environment of the stomach or neutral conditions found in the intestines, as well as specific cell or sub-cellular organelles [26]. To achieve a pH-stimulus response, it is crucial to ensure the stability of the PPEs at physiological pH, allowing for stable encapsulation of the drug until its release at the desired pH trigger location. Variations in pH disrupt these electrostatic interactions through protonation and deprotonation, leading to demulsification as a result of changes in particle wettability and subsequent drug release.

**Figure 9:**
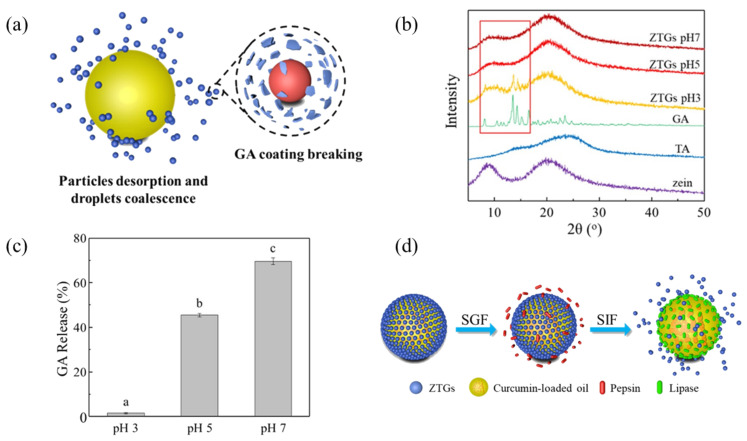
(**a**) Schematic diagram of ZTG-stabilized emulsions and ZTGs responsive to neutral pH. (**b**) XRD patterns of raw zein, TA, GA and ZTGs at pH values 3, 5 and 7. As shown in the red square, in the diffraction angle range of 10–20°, sample ZTGs have several sharp characteristic peaks of component GA at pH 3, whereas at pH 5 and 7 the characteristic peaks disappeared. (**c**) GA release from ZTGs at pH values 3, 5 and 7 in the aqueous phase. Different letters indicate significant differences (*p* < 0.05). (**d**) Schematic diagram of ZTG-stabilized emulsions responsive to SIF and SGF. Reproduced with permission from [39]. Copyright @2023 American Chemical Society.

Li et al. [39] prepared a gastrointestinal pH-sensitive Pickering emulsion using zein nanoparticles coated with bioactive glycyrrhizic acid (GA) to encapsulate and control the release of the hydrophobic drug curcumin. The pH-responsive behavior of these Pickering emulsions is driven by pH-dependent structural and solubility changes of GA on the particle surface. At pH 3, GA remains protonated and effectively coats the particles, ensuring emulsion stability. As the pH increases to 5–7, GA deprotonates, dissociates from the particle surface, and dissolves into the aqueous phase, as evidenced by the disappearance of specific XRD peaks and increased GA release (Figure 9). This shedding alters the particle wettability, disrupting their interfacial arrangement on oil droplets and inducing demulsification. Furthermore, GA transitions from rod-like micelles (pH 5–6) to monomers (pH > 7), further destabilizing the emulsion. These pH-mediated changes in GA solubility and particle surface properties facilitate the design of pH-sensitive Pickering emulsions for controlled release via pH-triggered demulsification. The pH-responsive behavior of the emulsion enhances the bioaccessibility of curcumin for oral administration. The therapeutic effects against cancer cells in vitro using the HCT-116 cell as a tumor model indicated that the pH-triggered co-delivery of the anti-cancer drugs realized the synergistic cancer therapy [71].

Furthermore, thermosensitive Pickering emulsions can be designed to exploit the temperature difference between the constant human body temperature (~37 °C) and the drug storage temperature [147]. Drug release can also be remotely controlled using applied magnetics and electric fields [148,149]. Also, photoresponsive materials such as azobenzene and coumarin moieties, which can change particle wettability upon irradiation, provide another strategy to control drug release. However, UV light-responsive release in DDS is limited by the low transdermal efficiency of light. In contrast, near-infrared (NIR, 650–950 nm) as an alternative external light source can penetrate deeper into tissues, offering greater promise for non-invasive stimulus-responsive delivery [150]. Multi-stimulus-responsive PPEs provide additional opportunities for precise control over drug release. With the development of interfacial property modulation, stimulus responsiveness and the introduction of new functionalities of particles, innovative DDS based on PPEs, such as time-space assembled droplets, stimulus-responsive phagocytosis droplets and droplet-responsive micro-robotics, are anticipated.

PPEs have the potential to serve as effective and controllable drug delivery systems, with model drug release being contingent upon the disruption of the Pickering emulsions. All the aforementioned stimulus-responsive strategies involve the rupture, melting, or deformation of particle structures, which may compromise the mechanical properties and stability of the emulsions. Consequently, it is essential to achieve a balance between the stability and release functionalities of stimulus-responsive Pickering emulsions. Given that the effectiveness and robustness of Pickering emulsion delivery systems are influenced by particle homogeneity, managing the heterogeneity of particle interstitials, as well as the stimuli-responsiveness and drug release behavior, remains a significant challenge in the research of Pickering emulsion DDS.

## 5. Conclusions

Recent research has demonstrated that protein-based particles serve as effective stabilizers in Pickering emulsions due to their tunable self-assembly properties and multi-level structures, making them suitable carriers for the encapsulation and delivery of both hydrophobic and hydrophilic drugs. The properties of protein particles can be optimized through various preparation methods and functionalization modifications, including adjustments in size, charge and wettability. These characteristics collectively influence the adsorption behavior of the particles at the oil–water interface and the subsequent delivery processes of PPEs. Numerous strategies and biomass sources have been explored to modify the surface characteristics and improve the emulsifying properties of protein particles.

The stability and release characteristics of the developed carriers can be fine-tuned by optimizing the Pickering emulsion formulation and processing parameters. Here, we highlight recent advancements in PPEs and their applications in drug delivery, focusing on strategies to stabilize Pickering emulsions from the perspective of particle interfacial adsorption properties based on advanced characterization techniques and simulation calculations, as well as the mechanisms to improve drug delivery efficiency. By rational designing interfacial effects, significant progress would be achieved in next-generation drug delivery systems.

Future research should focus on elucidating the release mechanism of PPEs delivering drugs. Particular attention should be given to PPE delivery systems with multiple structures (e.g., W/O/W emulsions), regarding the stabilization mechanism that enhances co-delivery bioavailability and the stimulus responsiveness of controlled release through the interplay of multiple structures and synergistic effect relationships, in particular towards in vitro and in vivo behavior. Furthermore, more studies are needed to address the current challenges associated with the industrial-scale development of these carriers and their applications in real pharmaceutical systems.

## Figures and Tables

**Table 1:** Different preparation methods of protein particles for stabilizing Pickering emulsion.

Preparation Approaches	Advantage	Limitation	Methods	Principles	Reference
Physical approaches	The common methods, simple operation, safe preparation, cheap cost and no new chemical formation	The particle structures stability dependent on environmental conditions, tends to dissociate into polymeric structures at the emulsion droplet interface upon addition, dilution or pH change	Mechanical methods	Micronization of particles via high shear, ball milling, sonication and supercritical fluid technology	[86]
			Isoelectric point methods	Proteins at the emulsion droplet interface aggregate to form nanoparticle as the pH is adjusted to the isoelectric point	[87]
			pH-cycle methods	In alkaline or acidic conditions, proteins first dissolve and then self-assemble into particles when the pH is adjusted to the level at which the protein becomes insoluble	[88]
			Antisolvent precipitation methods	Particle formation via supersaturation, nucleation, growth and agglomeration of the protein solutions by hydrogen bonding, electrostatic interaction and hydrophobic effects, including stepwise antisolvent precipitation methods and antisolvent coprecipitation methods	[89]
			Solvent evaporation methods	The water-insoluble proteins dissolve in a water-organic solvent mixture and spontaneously form particles by supersaturation as the organic solvent evaporates	[90]
			Heat-induced aggregation methods	Heat-treat proteins above denaturation temperature to cause denaturation, partial unfolding, and aggregation into particles, improve interfacial and emulsifying properties	[91]
			Non-electrostatic complexation methods	Mix two or more substances in aqueous protein solutions to form particles via non-electrostatic interactions, such as hydrophobic interactions and hydrogen bonding	[92]
Chemical approaches	The covalently bonded particles show more stable shape, solubility, thermal stability, and emulsifying properties	Certain toxic crosslinkers compromise the safety of the particle within the human body	Ionic crosslinking	Mix polymers with opposite charges or use divalent ions (e.g., Ca^2+^) to shield negative charges, adjust pH between protein isoelectric points to induce self-assembly, form complex particles through electrostatic interactions	[93]
			Heat-induced disulfide crosslinking methods	Heat-treat proteins to denature and aggregate, reduce free sulfhydryl groups and form strong intraparticle disulfide bonds	[94]
			Aldehyde-induced covalent crosslinking methods	Use aldehyde agents (e.g., glutaraldehyde) to crosslink free amino groups in proteins, strengthen nanoparticle integrity, enhance storage and emulsion stability	[95]
			Genipin-induced covalent crosslinking methods	Use genipin to crosslink free amino groups in proteins or protein-polysaccharide conjugate, form stable particles with low cytotoxicity and long-term stability	[96]
			Polyphenol-induced covalent crosslinking methods	Use oxidized polyphenols to covalently modify protein nanoparticles, enhance interfacial behavior, oxidative stability, and emulsion resistance to digestion, improve emulsion stability and nutrient retention	[97]
Biological approaches	High specificity, mild reaction conditions, and sustainable enzymes, improve functional properties and emulsion stability	Require further structural analysis to confirm particle formation and understand stabilization mechanisms, with limited enzyme types (e.g., transglutaminase, laccase) currently applied for crosslinking	Enzymatic methods	Use bottom-up methods to form nanoparticles by physical or ionic crosslinking followed by enzymatic covalent crosslinking, or use top-down methods to crosslink proteins into gels and mechanically break them into nanoparticles for Pickering emulsion stabilization	[98]

**Table 2:** Summary of different proteins for stabilizing Pickering emulsion.

Source of Protein	Type of Protein	Preparation Method	Emulsification Quantitative Parameters	Reference
Vegetable protein	Zein	Antisolvent precipitation	The Pickering emulsion was stable for 10 days at 1.5 wt% particle concentration and 50% oil content at room temperature.	[71]
	Soy protein	pH-induced crosslinking	Emulsions with 1.0–3.5 wt% nanoparticle concentrations and 50–70% oil fractions were stable at 4 °C for 20 days.	[102]
	Peanut protein	Electrostatic adsorption	Emulsion stabilized with peanut protein complex (containing 0.35 g/100 mL cellulose nanocrystals) exhibited good stability with low creaming index at 30% oil fractions after 30 d storage.	[103]
	Lupin protein	pH-induced precipitation	Heating greatly improved the emulsification performance of protein particles at high concentrations (>3%, *w*/*v*), and the emulsions were highly stable over 14 days storage. At a 0.5% protein concentration, it was not sufficient to stabilize the droplets.	[91]
	Gliadin	Antisolvent precipitation	Stabilization of an O/W emulsion at a low concentration (0.1%, *w*/*v*), showed the greatest resistance to coalescence and the most reduction in mean particle diameter over a 30-day storage period.	[104]
	gluten protein	Antisolvent precipitation	Emulsions with 4% glycosylated gluten nanoparticles and 40% oil-water volume showed the best thermal and storage stability (30 d).	[105]
Animal protein	β-lactoglobulin	Electrostatic interaction (polysaccharide–protein complexes)	At a controlled oil/water volume ratio of 1:9, concentrations of 0.8 wt% to 1.5% showed storage stability for up to 28 d, while the most severe creaming occurred in the emulsion stabilized by 0.2–0.8 wt% complexes.	[106]
	Casein	Glutaraldehyde covalent modification	Emulsions showed low instability index values, consistently below 0.2, indicating minimal aggregation or flocculation	[107]
	Ovalbumin	Acid-heat modification	Oleogel-based Pickering emulsions at low concentrations (10 or 20 mg/mL) displayed ultra stability over 90-day storage and outstanding freeze-thaw stability.	[108]

**Table 3:** Natural substances for modification protein particles to stabilize Pickering emulsion.

Natural Substances	Advantage	Principles	Preparation Method	Application	Reference
Protein-polysaccharide	Enhance hydrophilicity, interfacial properties and emulsion stabilityImprove emulsification, foaming, solubility and surface activity	Glycosylation of protein particles with polysaccharide via Maillard reaction between the reducing carbonyl group of the carbohydrate and the free amine group of the protein	Covalent bonding	WPI-Dextran (DX) conjugate microgel particles stabilize Pickering emulsion via Maillard reaction, delaying interfacial gastric proteolysis	[100]
		Modification of protein by non-covalent interactions with polysaccharide (e.g., electrostatic, hydrophobic, hydrogen bonding, van der Waals) to form soluble coacervates or insoluble aggregates	Non-covalent mediated	ZN-CS particle-stabilized Pickering emulsion delivery of quercetin, improving encapsulation efficiency and oral bioavailability	[109]
Protein-protein	Improve interfacial absorption and emulsion stability, increase storage modulus and viscosity	Fabricate protein-protein complex particles by assembling oppositely charged proteins via electrostatic interactions, hydrogen bonds, and hydrophobic interactions under low ionic strength, enhance solubility and stability through pH-shifting or enzymatic crosslinking	Electrostaticinteraction	Zein-propylene glycol alginate-whey protein microgel particles stabilized Pickering emulsion for delivery of β-carotene, improving the photothermal stability and storage stability, delaying the lipolysis during gastrointestinal digestion	[110]
Protein-phenolic	Enhance hydrophilicity, thermal stability, antioxidant activity, and emulsion stability, prevent protein aggregation	Fabricate protein-phenolic complex/composite particles by forming covalent bonds (via oxidation and nucleophilic reactions) or non-covalent interactions (e.g., hydrogen bonding, van der Waals forces)	Electrostaticinteraction	Pea protein-proanthocyanidin complex particles held together by hydrogen bonding, stabilized Pickering emulsion for proanthocyanidins delivery, increasing the storage stability	[101]

## Data Availability

No new data were created or analyzed in this study. Data sharing is not applicable to this article.
